# Retraction: Embracing the digital revolution: Examining the relationship between ICT adoption and carbon emissions in the Persian Gulf

**DOI:** 10.1371/journal.pone.0334865

**Published:** 2025-10-23

**Authors:** The PLOS One Editors

The *PLOS One* Editors retract this article [1] because it was identified as one of a series of submissions for which we have concerns about potential manipulation of the publication process, peer review integrity, and authorship. These concerns call into question the validity and provenance of the reported results. We regret that the issues were not identified prior to the article’s publication.

ZUR did not agree with the retraction. HS either did not respond directly or could not be reached.
